# Roentgen's Discovery

**Published:** 1896-05

**Authors:** 


### ARTICLE X.

### ROENTGEN'S DISCOVERY.

### HOW IT CAN BE APPLIED TO MEDICINE AND SURGERY.

The New York Medical News prints the following article by Henry W. Cattell, M. D., demonstrator of morbid anatomy in the University of Pennsylvania, explaining the application of Roentgen's discovery to medical science:

"Roentgen's weird and wonderful discovery is destined to enrich medicine with possibly the most valuable diagnostic process which recent years have witnessed. Within a month from Roentgen's first announcement sufficient experiments have confirmed the value of his discovery and have pointed toward applications which, though yet unachieved, only await a development of details similar to that which converted Daguerre's slow, faint and uncertain methods into the instantaneous and brilliant photography of the present day.

"In brief, Roentgen has discovered that the electrical current passed through a Ruhmkorff coil and thence to a Crookes vacum-tube, developes from the cathode an invisible form of energy having the double property of penetrating certain ordinarily opaque substances and of affecting an ordinary photographic plate. Flesh, for instance, is translucent to these rays, and bone and metal, glass and graphite are comparatively impervious. On this difference in penetrability depend the effects which are permanently recorded by ordinary photographic plates. Perfect clearness of definition has not yet been attained. It will be convenient to coin the words skiagraph or skotograph to characterize pictures produced by the X rays and to indicate at once their relationship to and their distinction from photographs.

"The surgical imagination can pleasurably lose itself in devising endless applications of this wonderful process. If it becomes possible to drive these mysterious rays through the entire body as clearly as they now penetrate the hand, the realm of utility will be practically boundless. It is stated that stone in the kidney has already been determined, and the opacity of glass has led to the detection of small pieces adherent to bone after accident. The similar opacity of lead may render the probe useless in gunshot wounds, except in rare cases, as when the bullet is buried in bone. A new means of distinguishing luxations from fractures is now added to the long list at our command. Obstetricians will readily perceive the immense value of to see the fœtus in utero after ossification of its bones has occurred. The representation of deformed pelvis in the living subject, of spondylisthesis, of calcareous infiltration of various parts, such as arteries, and of exostosis; all this opens up a tempting and promising field for practical research.

"Incredible as all this may seem the skiagraphs (or pictures) photographically reproduced show a number of objects selected at random.

The easy application of Roentgen's method of taking a picture on a sensitized plate renders its use at once possible in hospitals. The entire cost of such an apparatus need not exceed $50, and the expenditure of this amount will shortly be materially diminished. Doubtless the scientific and commercial ingenuity now being focused on this process will soon produce outfits at once simple, convenient and portable, and it is safe to say that most practitioners will then provide themselves with the means of seeing into the mysterious recesses of the body now accessible only by the means of the knife.

### THE PROCESS ANALYZED.

The News also prints an article by Prof. Goodspeed which gives an authentic description of the process from the standpoint of a physicist as follows:

"Probably never before has the entire scientific world been simultaneously aroused to such a pitch of excitement as that caused by the recent remarkable discovery of Prof. Roentgen, of Wursburg, that it is possible to produce photographic effects through opaque substances, such as wood, ebolite, flesh and similar dense materials, while glass which we customarily regard as the most transparent of media, obstructs the passage of the cathodal ray.

"If we speak of light as that portion of radiation which is capable of affecting the eye, the new agent, or the X ray, as the discoverer calls it, is certainly not light, and 'photographic' is evidently not the word to apply to the effects produced.

"As it seems probable that this exhibition of energy is either a form of radiation or is intimately related to it, the term radiography might with propriety be applied to the process of producing the impressions.

"The earlier reports from abroad indicated that the use of two induction coils were necessary, and that the secondary of one be connected with the primary of the other, and that a Crookes 'radiant matter' tube be connected with the secondary of the latter. As glass proved comparatively opaque, it was deemed advisable to have a small aluminum window in the tube through which the rays could more easily pass. Later experiments, however, prove conclusively that these refinements are not needed, though without them a much longer exposure—perhaps an hour or more—is required. A single induction coil run by a few storage cells will excite an ordinary Crookes tube quite enough to produce most of the results yet published.

the induction coil in its ordinary form is familiar to every practitioner. A much larger coil is needed than is used for physiological stimulation, that which is being used in the experiments at the physical laboratory of the University of Pennsylvania being about 12 inches long and 4½ inches in diameter. It was constructed by Apps, of London, and possesses a primary resistance of about 0.3 ohm and a secondary resistence of about 3.200 ohms. The Crookes tube represents as complete a vacuum as it is possible to obtain, and is supposed to have an interior pressure of about one-millionth of an atmosphere. The negative electrode from which the X rays' start is an aluminum disc about a half inch in diameter. With the apparatus at present used by the writer an exposure of an hour or more was allowed. An ordinary photographic camera is used, having an aluminum shutter, which remains closed. The sensitive plates are entirely enclosed in the photo-holder, which is placed about five inches from the end of the Crookes tube in a horizontal position on the table. The subject to be radiographed is placed on the cover of the plate-holder. The plates are developed and fixed in the usual manner. Radiograms to any extent may be printed upon any sensitized paper."

